# Fascial Remodeling After Botulinum Toxin Injection in Post-Stroke Spasticity: A Retrospective Ultrasonographic Study

**DOI:** 10.3390/toxins18080330

**Published:** 2026-07-30

**Authors:** Betül Aydın, Münire Nazlı Höbek Başer, İpek Midi, Naime Evrim Karadağ Saygı, Özge Keniş Coşkun

**Affiliations:** 1 Department of Physical Medicine and Rehabilitation, Marmara University, 34722 Istanbul, Turkey; 2 Department of Neurology, Marmara University, 34722 Istanbul, Turkey

**Keywords:** botulinum toxin type A, post-stroke spasticity, fascia, ultrasonography, fascial remodeling, rehabilitation, muscle ultrasound, Modified Ashworth Scale, Fugl–Meyer Assessment

## Abstract

Post-stroke spasticity involves both neural and non-neural mechanisms, including structural alterations of muscles and surrounding fascia. Although botulinum toxin type A (BoNT-A) is widely used to treat focal spasticity, its effects on fascial tissue remain unclear. This retrospective observational study investigated longitudinal changes in fascial thickness after BoNT-A injection using ultrasonography. Thirty-seven patients with post-stroke spasticity were included. Fascial thickness was measured at baseline, 3 weeks, and 3 months after treatment in the injected muscle regions. Clinical outcomes were assessed using the Modified Ashworth Scale (MAS), Fugl–Meyer Assessment (FMA), and Brunnstrom stages. Repeated-measures analysis demonstrated significant reductions in fascial thickness across all evaluated regions. Pairwise comparisons showed significant decreases from baseline to week 3 and month 3, whereas continued reductions between week 3 and month 3 were observed only in the flexor digitorum superficialis fascia, the flexor digitorum superficialis–flexor digitorum profundus interfascial layer, and the deep brachialis fascia. MAS scores and upper extremity Fugl–Meyer scores improved significantly, whereas Brunnstrom stages remained unchanged. These findings suggest that the therapeutic effects of BoNT-A may extend beyond neural inhibition to include fascial remodeling. Ultrasonographic assessment of fascial thickness may represent a potential imaging biomarker for monitoring structural treatment responses in post-stroke spasticity.

## 1. Introduction

Stroke remains one of the leading causes of long-term disability worldwide, and post-stroke spasticity is among its most common and functionally limiting complications. Depending on the population studied and the duration of follow-up, spasticity develops in approximately 20–40% of stroke survivors and may substantially impair motor performance, restrict daily activities, and reduce quality of life [1,2,3]. Botulinum toxin type A (BoNT-A) has become the standard focal treatment for post-stroke spasticity and is widely used to reduce muscle overactivity, facilitate rehabilitation interventions, and improve functional outcomes [3,4].

Traditionally, spasticity has been considered a consequence of upper motor neuron lesions leading to hyperexcitability of the stretch reflex. However, accumulating evidence indicates that the clinical manifestations of chronic spasticity cannot be explained solely by neural mechanisms. Prolonged muscle overactivity and immobilization induce secondary structural adaptations within muscles and surrounding connective tissues, including fibrosis, extracellular matrix accumulation, shortening of muscle–tendon units, and alterations in tissue mechanical properties [5,6,7,8]. These peripheral changes contribute substantially to passive stiffness and resistance to movement and are increasingly recognized as important determinants of long-term disability after stroke [5,6].

Among the peripheral structures involved in these adaptations, fascia has emerged as a key component of the myofascial system. Fascia forms a continuous connective tissue network that surrounds and interconnects muscles, tendons, nerves, and vascular structures. Beyond its traditional role as a passive supporting tissue, fascia plays an important role in force transmission, proprioception, intermuscular coordination, and biomechanical regulation [9,10,11,12]. Experimental and imaging studies suggest that chronic abnormal loading conditions may induce fascial thickening, fibrosis, and changes in extracellular matrix composition, ultimately affecting tissue mobility and mechanical behavior [9,10,12]. Recent advances in musculoskeletal ultrasonography have enabled non-invasive visualization and quantitative assessment of fascial morphology, creating new opportunities to investigate the contribution of fascial tissue to neurological disorders [9].

Emerging ultrasound studies have demonstrated that patients with chronic post-stroke spasticity exhibit significantly thicker fascia on the affected side compared with the unaffected side. Choi et al. reported substantial thickening of both crural and epimysial fascia in chronic stroke survivors with spasticity, while muscle stiffness differences were comparatively limited, highlighting the potential importance of fascial remodeling in the pathophysiology of spasticity [13]. Similarly, Magat et al. demonstrated increased fascial thickness and altered muscle architecture in spastic-paretic limbs, further supporting the concept that connective tissue adaptations may represent an important non-neural component of post-stroke motor impairment [10]. Collectively, these findings suggest that fascial remodeling may contribute to the persistence of motor dysfunction even when neural factors are partially controlled [13].

Although the effectiveness of BoNT-A in reducing spasticity is well established, its effects on fascial tissue remain poorly understood. The primary mechanism of BoNT-A involves inhibition of acetylcholine release at the neuromuscular junction, resulting in temporary chemodenervation and reduced muscle overactivity [3,4]. However, reduction in sustained muscle contraction may also alter the mechanical environment of surrounding connective tissues. Consequently, BoNT-A may influence fascial remodeling indirectly through changes in tissue loading, movement patterns, and muscle–fascia interactions. Previous imaging studies have mainly focused on muscle architecture, muscle volume, or muscle stiffness following BoNT-A treatment [14], whereas longitudinal investigations specifically evaluating fascial adaptations remain scarce. Therefore, the temporal evolution of fascial morphology after BoNT-A injection remains largely unknown.

A better understanding of fascial responses to BoNT-A treatment may provide novel insights into the non-neural mechanisms of spasticity and help identify imaging biomarkers associated with therapeutic response. Therefore, the primary aim of the present study was to investigate longitudinal changes in fascial thickness following botulinum toxin injection in patients with post-stroke spasticity using ultrasonographic assessment. A secondary aim was to explore the relationship between fascial remodeling and clinical outcomes, including spasticity severity and motor function. We hypothesized that botulinum toxin injection would result in progressive reductions in fascial thickness and that these structural changes would be associated with improvements in motor function and spasticity severity.

## 2. Results

A total of 37 patients were included. The mean age of the patients was 60.1 ± 12.2. The mean time since stroke was 71.9 ± 65.5 months. 48.6% of the patients had involvement on the right side. Other demographic properties are summarized in Table 1.

Clinical outcomes are summarized in Table 2. Upper extremity Fugl-Meyer scores demonstrated a significant improvement over time (overall *p* < 0.001). No significant temporal changes were observed in proximal upper extremity, distal upper extremity, or lower extremity Brunnstrom stages. In contrast, MAS scores significantly changed over time in all injected muscle groups (all *p* < 0.001). Post hoc analyses demonstrated significant reductions in MAS scores at week 3 compared with baseline, whereas no significant differences were observed between baseline and month 3 or between week 3 and month 3 after Bonferroni correction.

Repeated-measures ANOVA demonstrated significant temporal changes in fascia thickness in all evaluated regions (all overall *p* ≤ 0.003). Post hoc analyses with Bonferroni correction showed significant reductions between baseline and week 3 and between baseline and month 3 in all regions. Further reductions between week 3 and month 3 were observed in the FDS fascia, FDS-FDP interfascial fascia, and brachialis deep fascia, whereas no significant additional change was found in the pronator superficial fascia, pronator deep fascia, brachialis superficial fascia, or gastrocnemius fascia. These results are summarized in Table 3.

Significant positive correlations were observed between changes in superficial pronator fascia thickness and MAS at both week 3 (ρ = 0.561, *p* = 0.019) and month 3 (ρ = 0.485, *p* = 0.048). Similarly, changes in gastrocnemius fascia thickness were positively correlated with changes in MAS at week 3 (ρ = 0.456, *p* = 0.043) and month 3 (ρ = 0.581, *p* = 0.007). No significant correlations were identified between changes in fascia thickness and MAS scores in the finger flexor or brachialis muscles. Within the same muscle, changes in different fascial layers demonstrated strong positive correlations. Superficial and deep brachialis fascia thickness changes were strongly correlated at both week 3 (ρ = 0.786, *p* = 0.001) and month 3 (ρ = 0.825, *p* < 0.001). Likewise, superficial and deep pronator fascia thickness changes were significantly correlated at week 3 (ρ = 0.548, *p* = 0.023) and month 3 (ρ = 0.679, *p* = 0.003). In addition, fascial thickness changes remained highly consistent between week 3 and month 3 within the same fascial compartment for the brachialis, pronator, finger flexor (FDS and FDS–FDP interfascial fascia), and gastrocnemius muscles. Correlation analyses between changes in fascia thickness and muscle-specific MAS scores are summarized in Table 4.

## 3. Discussion

The present study investigated longitudinal changes in fascial thickness following botulinum toxin type A (BoNT-A) injection in patients with post-stroke spasticity using ultrasonographic assessment. The principal findings were fourfold. First, significant reductions in fascial thickness were observed in all evaluated fascial regions after treatment. Second, although all fascial layers demonstrated an early decrease following injection, the temporal pattern of remodeling differed among anatomical regions. Third, while significant improvements in spasticity were observed following treatment, the relationship between fascial remodeling and clinical improvement was not uniform across all muscles and fascial compartments. Finally, fascial thickness changes demonstrated high temporal consistency within the same fascial compartment, suggesting persistent structural remodeling following BoNT-A treatment.

Traditionally, post-stroke spasticity has been interpreted primarily as a consequence of abnormal supraspinal control leading to hyperexcitability of the stretch reflex. However, increasing evidence indicates that the clinical manifestations of chronic spasticity cannot be explained solely by neural mechanisms. Long-standing muscle overactivity induces progressive structural adaptations within muscles and surrounding connective tissues, including extracellular matrix accumulation, collagen deposition, fibrosis, and alterations in passive mechanical properties. These peripheral tissue changes contribute substantially to muscle stiffness, reduced joint mobility, and contracture formation, and may partly explain the discrepancy frequently observed between neurophysiological measures and clinical resistance to passive movement. Consequently, current concepts increasingly recognize spasticity as a disorder involving both neural and non-neural components, with connective tissue remodeling playing an important role in long-term functional impairment.

The mechanisms underlying the observed reductions in fascial thickness are likely multifactorial. Botulinum toxin type A (BoNT-A) exerts its primary therapeutic effect through inhibition of acetylcholine release at the neuromuscular junction, thereby reducing excessive muscle activation and muscle tone [3,8]. However, the resulting decrease in sustained muscle contraction may also modify the mechanical environment of the surrounding connective tissues. Fascia is continuously exposed to tensile forces generated by muscle activity, and persistent spastic contraction is thought to promote connective tissue remodeling through increased collagen deposition, extracellular matrix accumulation, and reduced tissue gliding [5,7]. By alleviating abnormal mechanical loading, BoNT-A may create a more favorable environment for partial reversal of these structural adaptations. Although the precise biological mechanisms remain to be fully elucidated, our findings support the concept that fascial tissue is a dynamic structure capable of responding to changes in mechanical loading rather than representing a static anatomical envelope [9].

Recent advances in fascial research have substantially changed the understanding of fascia from a passive connective tissue layer to an active component of the myofascial system. Experimental and anatomical studies have demonstrated that fascia contributes to force transmission, proprioception, intermuscular coordination, and biomechanical regulation [9,12]. Furthermore, fascial fibroblasts are highly responsive to mechanical stimuli and are capable of modifying extracellular matrix turnover in response to altered loading conditions [12]. Chronic immobilization or sustained muscle overactivity has been associated with increased collagen synthesis, reduced fascial gliding, and fascial thickening [10,11]. Therefore, the reductions in fascial thickness observed after BoNT-A injection in the present study may reflect adaptive remodeling secondary to restoration of a more physiological mechanical environment rather than a direct pharmacological effect of the toxin on fascial tissue. Taken together, these findings support the hypothesis that fascial remodeling represents a potentially modifiable non-neural component of post-stroke spasticity and may contribute to the clinical benefits observed following BoNT-A treatment.

Our findings are consistent with the growing body of evidence suggesting that fascial alterations constitute an important non-neural component of post-stroke spasticity. Previous ultrasonographic studies have demonstrated increased fascial thickness in spastic limbs compared with unaffected limbs or healthy controls, indicating that chronic neurological impairment is accompanied by connective tissue remodeling [13]. Similarly, alterations in muscle architecture and fascial morphology have been reported in patients with chronic spasticity, supporting the concept that prolonged abnormal muscle activity induces structural adaptations extending beyond muscle fibers alone. While these studies primarily provided cross-sectional evidence of fascial involvement, the present study extends the current literature by demonstrating that fascial thickness is not a static characteristic but changes longitudinally following botulinum toxin treatment.

An additional finding of our study was that the temporal pattern of fascial remodeling differed among anatomical regions. Whereas the superficial and deep fasciae of the pronator muscle, the superficial brachialis fascia, and the gastrocnemius fascia exhibited an early reduction followed by stabilization, the FDS fascia, the FDS–FDP interfascial layer, and the deep brachialis fascia demonstrated progressive thinning throughout the three-month follow-up period. These findings may reflect regional differences in fascial composition, mechanical loading, or muscle function. Deep fascial layers are subjected to complex multidirectional tensile forces and play an important role in force transmission between adjacent muscles. Consequently, they may require a longer period to adapt to changes in muscle activity after BoNT-A treatment. Although this hypothesis remains speculative, it provides a plausible explanation for the heterogeneous remodeling patterns observed across different muscle groups and warrants further investigation in prospective imaging and histological studies.

Another important finding of the present study was that the relationship between fascial remodeling and clinical improvement was not uniform across all evaluated muscles. Significant correlations between changes in fascial thickness and reductions in muscle-specific MAS scores were observed only in the superficial pronator fascia and gastrocnemius fascia, whereas no significant associations were identified in the finger flexor or brachialis fasciae. These findings suggest that structural fascial adaptation does not necessarily translate into measurable clinical improvement and that the relationship between fascial remodeling and spasticity reduction may depend not only on the injected muscle but also on the fascial compartment evaluated.

Interestingly, fascial thickness changes demonstrated remarkable temporal consistency within the same fascial compartment throughout the follow-up period. In addition, superficial and deep fascial layers of both the brachialis and pronator muscles exhibited highly coordinated changes over time. These findings suggest that fascial remodeling following BoNT-A represents a structured biological response rather than random measurement variability. Moreover, the persistence of reduced fascial thickness despite partial recovery of spasticity at month 3 may indicate that structural adaptation of fascial tissue outlasts the peak clinical effects of botulinum toxin. However, this hypothesis requires confirmation in prospective longitudinal studies.

This study has several limitations. First, because botulinum toxin treatment was individualized according to each patient’s clinical presentation and rehabilitation goals, multiple muscles were injected in most patients. Consequently, analyses were performed on a muscle-specific rather than a patient-specific basis. Although this approach precludes assessment of the independent contribution of individual injections and potential interactions between simultaneously treated muscles, it reflects routine clinical practice in the management of post-stroke spasticity. Second, the retrospective design and relatively small sample size within each muscle group may have limited the statistical power to detect associations between fascial remodeling and clinical improvement. Furthermore, ultrasonographic assessment provides morphological information only and cannot directly characterize the biological mechanisms underlying fascial remodeling. The retrospective nature of this study also hinders the ability to claim a standardized rehabilitation protocol was applied to the patients, so the effect of rehabilitation procedures during follow-up should also be kept in mind. Finally, follow-up was limited to three months after treatment. Therefore, the long-term persistence of fascial remodeling beyond the expected duration of the pharmacological effects of botulinum toxin remains unknown. In addition, the absence of a non-injected control group prevents differentiation between treatment-related fascial remodeling and spontaneous longitudinal changes. The retrospective design also hindered the ability to provide patients with a standardized rehabilitation protocol, to follow up on their participation, and to note the exact number of sessions attended. Prospective studies including larger patient populations, longer follow-up periods, and complementary imaging modalities such as elastography are warranted to further clarify the clinical significance of fascial adaptations after botulinum toxin treatment.

## 4. Conclusions

This study demonstrated that botulinum toxin type A treatment was associated with significant longitudinal reductions in fascial thickness in patients with post-stroke spasticity. These structural changes were accompanied by improvements in spasticity severity and upper extremity motor function, suggesting that the therapeutic effects of BoNT-A may extend beyond neural inhibition to include remodeling of the fascial tissues. The heterogeneous associations observed between fascial remodeling and clinical improvement suggest that structural responses to BoNT-A may differ across muscles and fascial compartments. Ultrasonographic assessment of fascial thickness may serve as a useful imaging biomarker for monitoring structural treatment responses. Further prospective studies with larger cohorts are warranted to clarify the biological mechanisms underlying fascial remodeling and its long-term clinical significance.

## 5. Material and Methods

### 5.1. Study Design and Participants

This retrospective observational study was conducted at the Department of Physical Medicine and Rehabilitation, Marmara University Pendik Training and Research Hospital, Istanbul, Türkiye. Medical records of patients with post-stroke spasticity who underwent botulinum toxin type A (BoNT-A) injection between July 2025 and April 2026 were retrospectively reviewed. The study was approved by the Marmara University Faculty of Medicine Ethics Committee, and all procedures were conducted in accordance with the Declaration of Helsinki.

**Figure 1:**
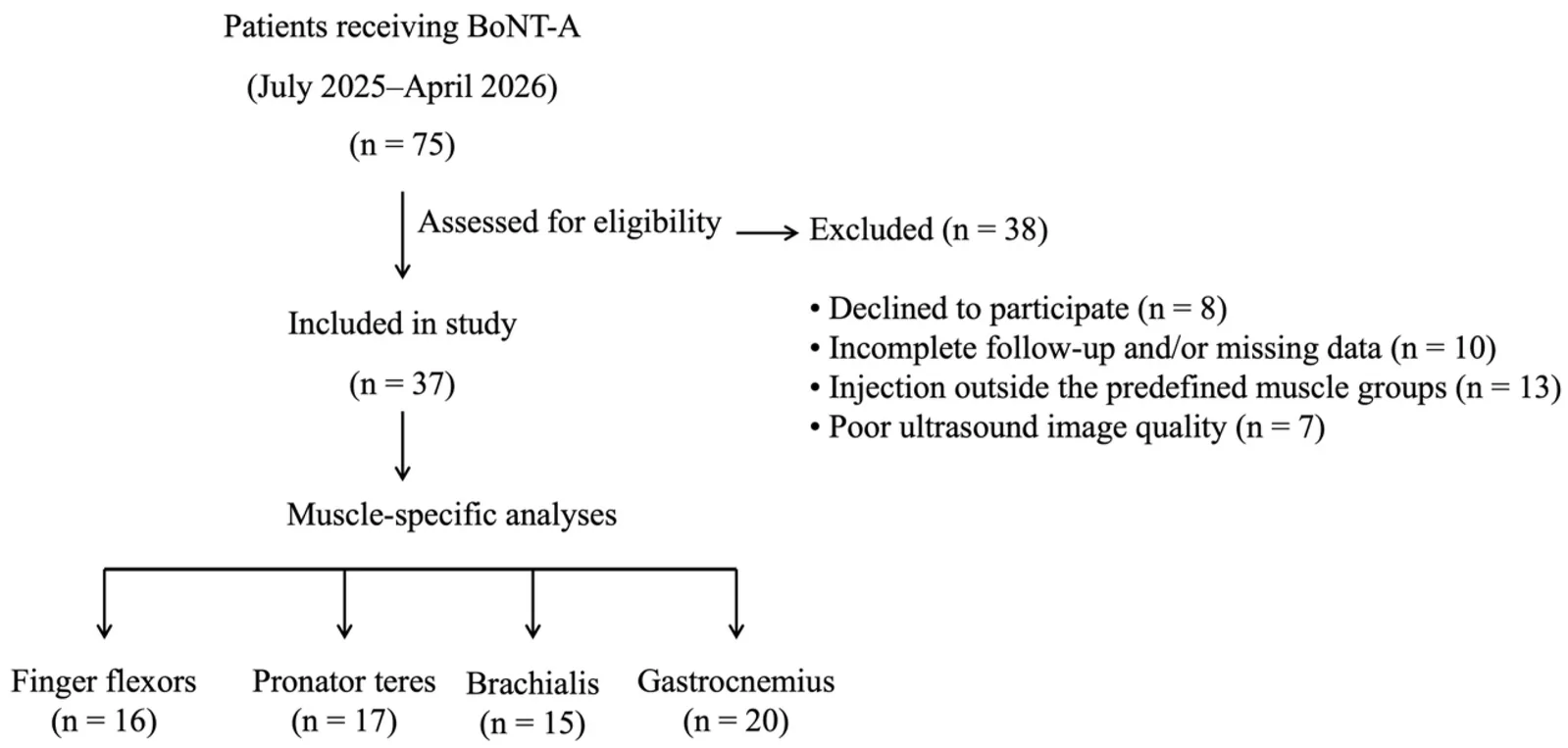
Patient flowchart.

Patients aged 18 years or older with ischemic or hemorrhagic stroke occurring at least six months before enrollment and scheduled for BoNT-A treatment for focal spasticity were eligible for inclusion. Additional inclusion criteria were the availability of complete demographic and clinical data and the absence of connective tissue or dermatological disorders that could affect fascial morphology. Patients with major upper or lower extremity musculoskeletal injuries (e.g., tendon rupture, fracture, or tendinopathy), severe psychiatric disorders, incomplete follow-up data, or missing ultrasonographic measurements were excluded from the study. The patients continued their personalized rehabilitation sessions that included range of motion and stretching exercises that target upper and lower extremities, balance training and walking exercises, strengthening exercises. Figure 1 shows the flowchart of patient selection, number of muscles injected, and muscles and related fascia investigated.

### 5.2. Botulinum Toxin Injection Protocol

Botulinum toxin type A (Botox^®^, Allergan Inc., Irvine, CA, USA) was administered for the treatment of focal post-stroke spasticity according to individualized clinical indications. The selection of target muscles and toxin doses was based on each patient’s pattern of spasticity, functional impairment, and rehabilitation goals. All injections were performed by a single experienced physiatrist using a combined ultrasound (US)- and electromyography (EMG)-guided technique to ensure accurate localization of the target muscles and precise toxin delivery. The brachalis, pronator teres, flexor digitorum superficialis, flexor digitorum profundus, medial head of the gastrocnemius, and soleus muscles were targeted. All these muscles were injected with a standard dose of 50 UI of botulinum toxin A [15].

### 5.3. Ultrasonographic Assessment

Ultrasonographic examinations were performed by a second experienced physician who was not involved in the injection procedure or clinical assessments prior to injection therapies. The examiner was blinded to all clinical evaluation results throughout the study. Fascial thickness was assessed using an Esaote ultrasound system equipped with a 12 MHz linear-array transducer.

**Figure 2:**
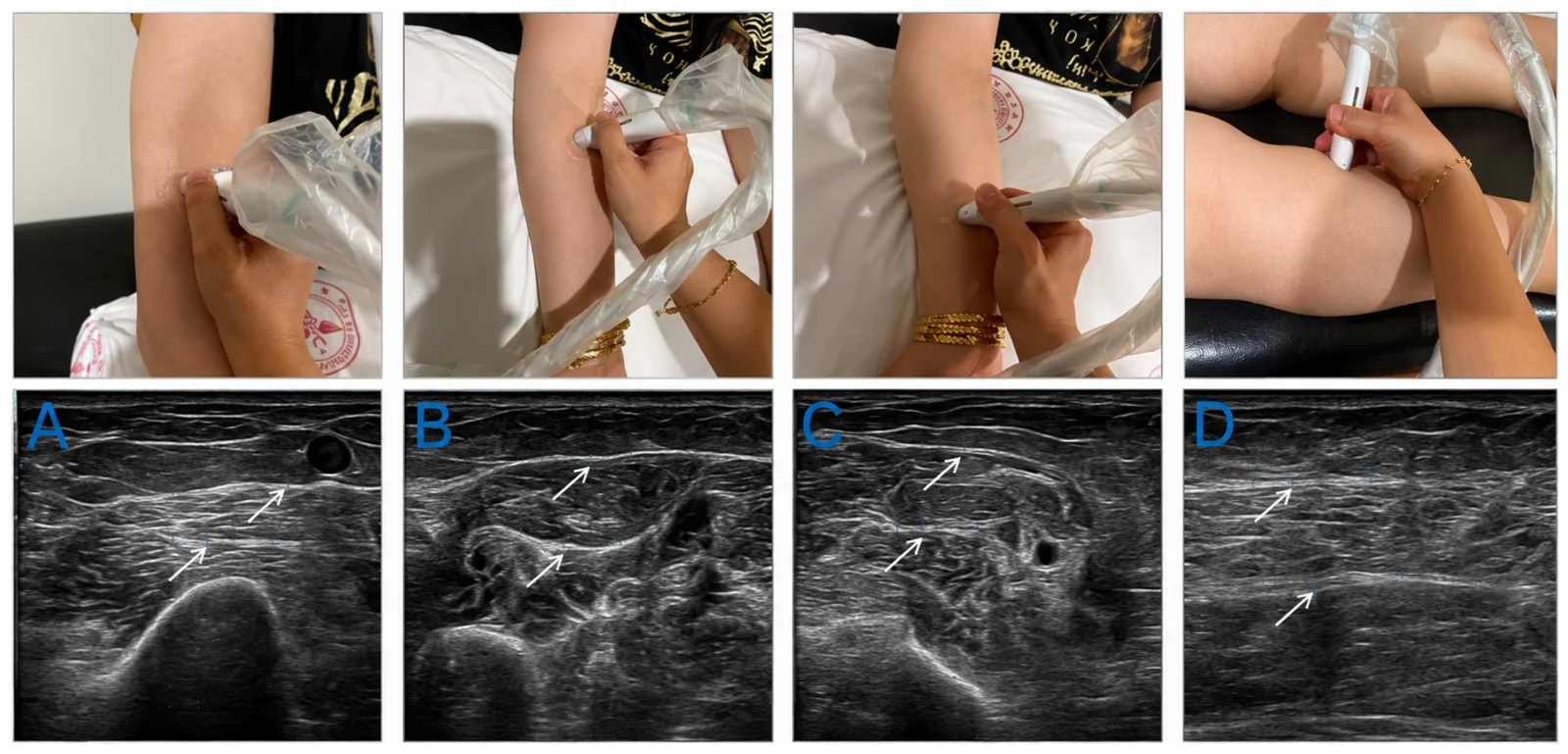
Ultrasound probe placement at standardized anatomical landmarks (**upper row**) and representative transverse B-mode ultrasound images (**lower row**) demonstrating fascial thickness measurements. (**A**) Brachialis fascia. (**B**) Pronator teres fascia. (**C**) Flexor digitorum superficialis/flexor digitorum profundus (FDS/FDP) fascia. (**D**) Medial gastrocnemius fascia. White arrows indicate the superficial and deep borders of the fascia. Blue marks show where the fascia was measured.

Measurements were obtained in the transverse plane at standardized anatomical landmarks in the injected muscle regions. The brachialis fascia was assessed at the anterior aspect of the mid-distal third of the humerus, the pronator teres fascia at the midpoint of the muscle belly in the proximal forearm, the fasciae of the flexor digitorum superficialis (FDS) and flexor digitorum profundus (FDP) at the mid-forearm level, and the medial gastrocnemius fascia at the site of maximal muscle thickness [16] (Figure 2).

Ultrasonographic assessments were performed immediately before BoNT-A injection (baseline), at 3 weeks, and at 3 months after treatment. Patients were allowed to continue their rehabilitation programs during follow-up, but the rehabilitation was not standard among the patients, considering their individual needs. Both superficial and deep fascial layers were evaluated when applicable. Three consecutive measurements were obtained from each fascial layer, and the mean value was used for statistical analysis to improve measurement reliability. All examinations were performed under standardized conditions with the muscles in a relaxed state. The measurements were made from the obtained images.

### 5.4. Clinical Assessment

Clinical evaluations were performed at baseline, 3 weeks, and 3 months after BoNT-A injection by experienced physicians using standardized assessment procedures. Spasticity severity was assessed for each injected muscle using the Modified Ashworth Scale (MAS), a six-point ordinal scale ranging from 0 to 4 (including grade 1+) that evaluates resistance to passive muscle stretching [17]. Motor impairment of the upper extremity was evaluated using the Fugl–Meyer Assessment (FMA), a validated stroke-specific measure of motor recovery with higher scores indicating better motor performance [18]. Motor recovery was further classified according to the Brunnstrom Recovery Stages for the upper and lower extremities, which describe sequential stages of neurological recovery after stroke [19].

### 5.5. Statistical Analysis

Statistical analyses were performed using IBM SPSS Statistics version 26.0 (IBM Corp., Armonk, NY, USA). Continuous variables were presented as mean ± standard deviation (SD), whereas categorical variables were expressed as frequencies and percentages.

Because botulinum toxin injections were individualized according to each patient’s clinical presentation, fascia thickness measurements were analyzed on a muscle-specific basis. Changes in fascia thickness over time (baseline, 3 weeks, and 3 months) were evaluated separately for each injected muscle using repeated-measures analysis of variance (ANOVA). When a significant main effect of time was detected, pairwise comparisons were performed using the Bonferroni correction. Other continuous variables measured at three time points were analyzed using repeated-measures ANOVA, whereas ordinal variables (MAS and Brunnstrom stages) were analyzed using the Friedman test with Bonferroni-adjusted post hoc comparisons. Since there were three consecutive measurements, a *p* value of <0.025 was considered significant.

Correlations between fascia thickness measurements and clinical parameters were assessed using Spearman’s rank correlation coefficient. Correlation analyses were considered exploratory. A two-sided *p* value < 0.05 was considered statistically significant.

## Figures and Tables

**Table 1:** Baseline characteristics of the study population (n = 37).

Variable	Value
Age (years), mean ± SD	60.1 ± 12.2
Female sex, n (%)	20 (54.1%)
Right-sided involvement, n (%)	18 (48.6%)
Time since stroke (months), mean ± SD	71.9 ± 65.5
Upper extremity Brunnstrom stage (proximal), mean ± SD	3.41 ± 0.96
Upper extremity Brunnstrom stage (distal), mean ± SD	3.68 ± 0.99
Lower extremity Brunnstrom stage, mean ± SD	3.80 ± 0.89
Upper extremity Fugl-Meyer score, mean ± SD	21.7 ± 12.1
Lower extremity Fugl-Meyer score, mean ± SD	18.9 ± 3.3
Gastrocnemius MAS, mean ± SD	2.50 ± 0.51
Brachialis MAS, mean ± SD	2.53 ± 0.52
Finger flexor MAS, mean ± SD	2.56 ± 0.51
Pronator MAS, mean ± SD	2.53 ± 0.51

Data are presented as mean ± standard deviation (SD) or number (%), as appropriate. Abbreviations: MAS, Modified Ashworth Scale.

**Table 2:** Clinical outcomes during follow-up.

Variable	Baseline	Week 3	Month 3	Overall *p*-Value
Upper extremity Fugl-Meyer score	21.73 (16.38–27.08)	22.86 (17.51–28.22)	22.36 (16.99–27.74)	<0.001
Upper extremity Brunnstrom stage (proximal)	3 (3–4)	3 (3–4)	3 (3–4)	1.000
Upper extremity Brunnstrom stage (distal)	4 (3–4.25)	4 (3–4.25)	4 (3–4.25)	1.000
Lower extremity Brunnstrom stage	3.5 (3–5)	4 (3–5)	3.5 (3–5)	0.368
Finger flexor MAS	3 (2–3)	2 (1–2)	2 (2–2)	<0.001
Pronator MAS	3 (2–3)	2 (1–2)	2 (2–2)	<0.001
Brachialis MAS	3 (2–3)	1 (1–2)	2 (2–2)	<0.001
Gastrocnemius MAS	2.5 (2–3)	2 (1.25–2)	2 (2–3)	<0.001

Note. Data are presented as estimated marginal means with 95% confidence intervals (95% CI) for Fugl-Meyer scores and as median (interquartile range [IQR]) for Brunnstrom stages and Modified Ashworth Scale (MAS) scores. Fugl-Meyer scores were analyzed using repeated-measures ANOVA, whereas Brunnstrom stages and MAS scores were analyzed using the Friedman test.

**Table 3:** Longitudinal changes in fascia thickness following botulinum toxin injection.

Fascia (n)	Baseline Mean (95% CI)	Week 3 Mean (95% CI)	Month 3 Mean (95% CI)	Overall *p*	Baseline vs. Week 3	Baseline vs. Month 3	Week 3 vs. Month 3
FDS fascia (n = 16)	0.758 (0.684–0.831)	0.671 (0.629–0.713)	0.652 (0.612–0.692)	0.001	0.006	0.003	0.006
FDS-FDP interfascial fascia (n = 16)	0.741 (0.666–0.817)	0.622 (0.588–0.655)	0.609 (0.580–0.637)	<0.001	0.001	<0.001	0.011
Pronator superficial fascia (n = 17)	0.788 (0.717–0.858)	0.702 (0.660–0.743)	0.692 (0.648–0.737)	<0.001	0.001	0.001	0.252
Pronator deep fascia (n = 17)	0.742 (0.685–0.798)	0.647 (0.602–0.692)	0.643 (0.598–0.687)	<0.001	<0.001	<0.001	1.000
Brachialis superficial fascia (n = 15)	0.770 (0.706–0.834)	0.688 (0.645–0.731)	0.686 (0.640–0.732)	0.001	0.001	0.004	1.000
Brachialis deep fascia (n = 15)	0.647 (0.596–0.697)	0.605 (0.575–0.635)	0.591 (0.560–0.622)	0.003	0.014	0.008	0.028
Gastrocnemius fascia (n = 20)	0.759 (0.708–0.810)	0.677 (0.628–0.725)	0.685 (0.638–0.731)	<0.001	0.001	0.002	0.813

Data are presented as estimated marginal means with 95% confidence intervals (CI). Overall *p*-values were obtained using repeated-measures analysis of variance (ANOVA). Greenhouse–Geisser correction was applied when the assumption of sphericity was violated. Pairwise comparisons were adjusted using the Bonferroni correction.

**Table 4:** Correlations between changes in fascia thickness and muscle-specific MAS scores.

Muscle	Fascia	Week 3 ΔMAS ρ (*p*)	Month 3 ΔMAS ρ (*p*)	Temporal Consistency Week 3 vs. Month 3 ρ (*p*)
Finger flexor	FDS superficial fascia	0.000 (1.000)	0.275 (0.304)	0.977 (<0.001)
	FDS–FDP interfascial fascia	0.431 (0.095)	0.287 (0.281)	0.983 (<0.001)
Pronator	Superficial fascia	0.561 (0.019)	0.485 (0.048)	0.968 (<0.001)
	Deep fascia	−0.056 (0.831)	−0.342 (0.179)	0.934 (<0.001)
Brachialis	Superficial fascia	−0.042 (0.880)	0.140 (0.619)	0.786 (0.001)
	Deep fascia	0.043 (0.880)	0.228 (0.413)	0.825 (<0.001)
Gastrocnemius	Deep fascia	0.456 (0.043)	0.581 (0.007)	0.882 (<0.001)

Note. Week 3 ΔMAS and Month 3 ΔMAS represent Spearman correlation coefficients (ρ) between changes in muscle-specific Modified Ashworth Scale (MAS) scores and changes in fascia thickness from baseline to week 3 and baseline to month 3, respectively. Temporal consistency represents the correlation between fascia thickness changes at week 3 and month 3 within the same fascial compartment.

## Data Availability

The original contributions presented in this study are included in the article. Further inquiries can be directed to the corresponding author.
